# Adaptation of a neutron radiography instrument for live cell irradiation

**DOI:** 10.1038/s41598-025-02382-4

**Published:** 2025-05-22

**Authors:** Nicholas Howell, Frederic Sierro, Raya Jarrah, Christopher Dobie, Joseph J. Bevitt, Ulf Garbe, Klaudiusz Jakubowski, Mitra Safavi-Naeini

**Affiliations:** 1 Australian Nuclear Science and Technology Organisation, Lucas Heights, NSW Australia; 2 University of Wollongong, Wollongong, NSW Australia

## Abstract

Neutron Capture Therapy (NCT) for cancer treatment is experiencing renewed interest due to advancements in accelerator-based neutron beams, treatment planning software, and patient positioning devices. This study presents the adaptation of an existing neutron radiography beamline (Dingo), at the OPAL research nuclear reactor, for radiobiological research and novel neutron capture agent development. Human glioblastoma cell cultures were irradiated for up to 10 min with a flux of 2.57 × 10^8^ n/cm^2^ s (± 2.73 × 10^7^) and the resulting impact was quantified by assessing DNA damage by both immunocytochemistry and flow cytometry. This low cost methodology extends the capability of an existing beamline to allow the development of novel neutron capture agents and study of neutron radiobiological mechanisms. Increasing availability of neutron sources for biological study in this fashion will accelerate the development of NCT for disease specific clinical application.

## Introduction

**Fig. 1:**
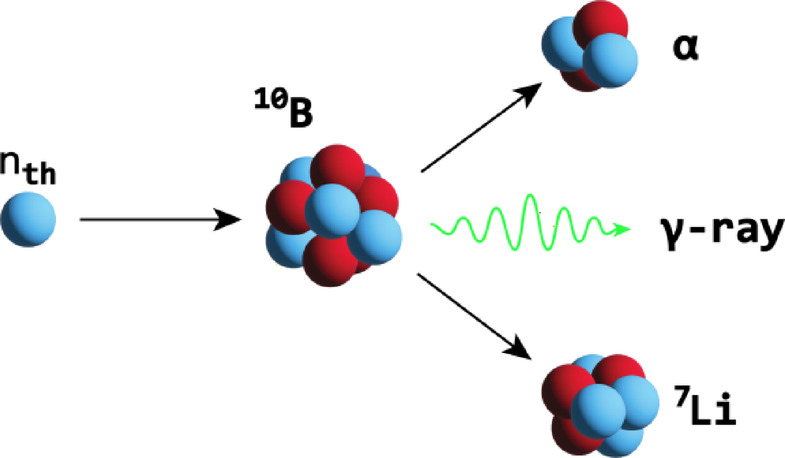
Schematics of the (n,α) neutron capture reaction in ^10^B.

Neutron capture therapy (NCT) for the treatment of cancer is experiencing a resurgence of interest. Advances in compact accelerator-based neutron sources with appropriate beam configuration systems have primarily driven this by separating the technique from costly and cumbersome fission-based sources, alongside improvements in treatment planning software and patient positioning devices^1^. Generally, NCT is an external beam radiotherapy modality that utilises neutron capture agents (NCA)—biochemically targeted stable isotopes of high neutron capture cross-section—in conjunction with an external neutron beam to trigger thermal neutron capture reactions within the target tissue. Current methods approved for clinical use deliver ^10^B isotope to cancerous tissue via metabolic accumulation^1^. The thermal neutron capture reactions of ^10^B (Fig. 1) are the foundational principle of boron neutron capture therapy (BNCT). The dominant reaction (n,α) results in the production of a high linear energy transfer (LET) α-particle (1.47 MeV/μm), recoil ^7^Li nucleus (0.84 MeV/μm) and a prompt γ-ray (478 keV), via the following mechanism^2^:

A second, lower abundance (6%), (n,α) reaction occurs resulting in a 1.77 MeV α-particle plus 1.015 MeV Lithium nuclei. A third possible reaction is a basic radiative capture (n,γ) whereby ^10^B absorbs a neutron, emitting a gamma ray and forming stable ^11^B. This particular reaction has a much smaller cross section compared to the (n,α) reaction cross section of 3840 barns. The particles’ produced by the (n,α) reaction have short penetration depth offering a precise targeting capability, causing lethal damage to the tumour or other targeted cells while sparing surrounding healthy tissue^3^.

In Japan, NCT has been approved for treating recurrent head and neck cancers, and promising results have been observed in other cancers like melanoma and brain tumours^4^.

Additionally, recent work has demonstrated that biologically relevant and clinically useful neutron fluxes are generated internally during proton and heavy-ion particle therapy, as a by-product of the particle irradiation itself^5,6^. Referred to as Neutron Capture Enhanced Particle Therapy (NCEPT)^7^, this concept extends the principles of NCT to applications in particle therapy (proton and carbon ion) treatments. Utilising this internally generated thermal neutron field could reduce primary ion beam doses and associated fractions, potentially improving patient outcomes and quality of life while offering a new treatment option for certain poor-prognosis cancers.

In order to optimise and progress NCT research, access to thermal neutron radiation sources is essential. In general, the primary constraint for most radiotherapy basic research and development is accessing appropriate radiation sources. Access to the limited beam time available for research on clinical instruments is highly competitive and the costs associated with running these machines greatly limit the total time available. An inexpensive, straightforward neutron irradiation system that minimally affects standard operations would enable efficient and iterative experimentation necessary for developing novel pharmaceuticals and research of radiobiological mechanisms. This manuscript describes the repurposing of a pre-existing neutron imaging instrument (referred to as *Dingo*)^8,9^, in use on the Open-Pool Australian Lightwater (OPAL) research nuclear reactor run by the Australian Nuclear Science and Technology Organisation, for the purposes of radiobiological research and novel NCT development. Dingo is currently the only thermal neutron beam available for research in Australia.

Research nuclear reactors like OPAL operate within highly restrictive environments governed by multiple regulatory frameworks and safety protocols. These facilities must comply with stringent national nuclear regulatory bodies (such as ARPANSA in Australia) that enforce comprehensive licensing requirements and regular safety assessments. Radiation safety considerations necessitate all movements of staff and equipment around the facility. Work health and safety protocols are exceptionally rigorous and detailed, requiring multiple levels of approval before being modified.The methodology described here has been designed to work within these multifaceted constraints and provide minimal disruption to regular operations.

The Dingo instrument is designed as a thermal neutron imaging instrument and its primary uses include palaeontology, materials science, archeology and industrial/engineering applications^8^. It is capable of neutron radiography and tomography and has two primary configurations:

1. High resolution mode: 1 cm diameter aperture producing a uniform field of 1.15 × 10^7^ n/cm^2^⋅s at the sample stage^10^.
2. High intensity mode: 2 cm diameter aperture producing 4.7 × 10^7^ n/cm^2^⋅s at the sample stage^10^.

**Fig. 2:**
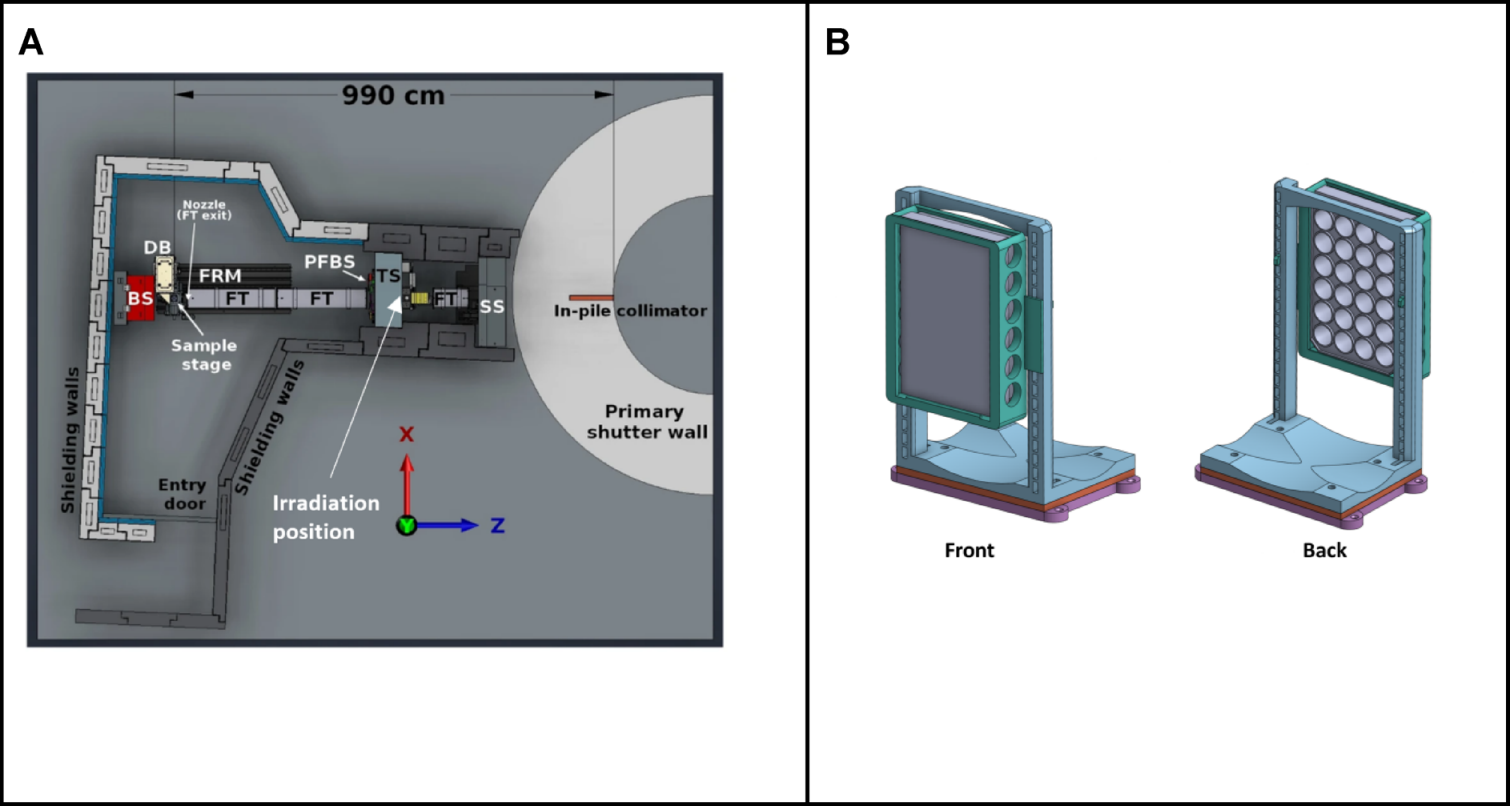
(**A**) Schematic diagram of the Dingo beamline^10^. SS = secondary shutter; TS = tertiary shutter; FT = flight tube; PFBS = pre-flight tube beam slits; FRM = floor rail mounting; DB = detector box; BS = beam stop. The relative positions of the sample stage and irradiation position are indicated. (**B**) Sample mount designed for holding tissue culture plates vertically positioned within the neutron beam at the irradiation position. Samples are loaded onto this mount before being driven up to the irradiation position immediately behind the tertiary shutter. The modular design allows for various sample formats to be mounted and positioned for irradiation.

Even when operating in high-intensity mode the neutron flux at the sample stage is approximately 10 times lower than the minimum recommendation of 5 × 10^8^ n/cm^2^⋅s for reactor based BNCT^11,12^. The sample stage used for imaging sits 9.9 m from the primary shutter (Fig. 2). The inverse square law states that the flux of neutrons decreases proportionally to the square of the distance from the source, indicating that halving the distance between the primary shutter and the sample could achieve a 4 × increase in flux on the target. This would increase the flux to ~ 2 × 10^8^ n/cm^2^ s, with further gains possible the closer to the primary shutter the sample can be located^13^. These increases, while still lower than the minimum recommended for BNCT, put the flux within a range that is usable for in vitro radiobiological and NCA structure–activity studies.

An accessible location, adjacent to the tertiary shutter, was identified as a viable irradiation location. This positions the samples ∽4.8 m from the primary shutter, reducing the distance by ~ 51%, potentially generating the required increase in flux. As no sample stage is present at that location a mechanism to move the sample up to the tertiary shutter has to be developed. This system must consistently and effectively position viable cell cultures at this location—situated at the beam path entrance to the instrument hutch across the 0.5 m thick concrete shielding wall. The tunnel passing through this wall between the two helium flight tubes measures 150 mm in width and 300 mm in height.

When repurposing existing infrastructure for radiobiology, successful live cell irradiation has three major requirements:

1. The beam must be reliably located and its shape determined to allow effective targeting.
2. The thermal neutron flux must be calculated so irradiation times can be determined and the fluence on the sample recorded.
3. Cells must be placed into the path of the beam, while maintaining sterile conditions and viability, in a format suitable for the desired downstream analysis, for the required period of time.

An enabling factor of this work has been the successful development of a fully validated Geant4 Monte Carlo simulation model of the Dingo beamline^10^. This in silico model simulates the entire beampath and allows precise prediction of the neutron field. This model is essential for ensuring the efficient use of the beam for irradiation purposes. This model has been validated in-beam and out-of-beam using a combination of absolute and relative measurements, including neutron activation analysis and Bonner sphere spectroscopy^14^. These are now able to be employed for quality assurance and validation purposes during radiobiological experimentation. The primary technique used for neutron flux measurements on the beamline is neutron activation analysis (NAA) of high-purity bare and cadmium covered gold foils. Gold responds to both thermal and epithermal neutrons, with significant cross-section peaks of 2.7 × 10^5^ barn and 3.6 × 10^3^ barn at 4.9 eV and 60.3 eV respectively, plus several resonance peaks in the hundreds of barns. To isolate the thermal neutron flux measurement, gold is typically used alongside cadmium-covered foil or wire because cadmium effectively absorbs thermal neutrons. This allows the epithermal neutron capture reaction rate from the cadmium measurement to be subtracted from the bare gold measurement, thereby isolating the thermal neutron flux^15^.

To achieve these irradiation conditions a method of using radiochromic films was employed to locate and target the beam while flux was measured by way of gold neutron activation analysis. A sample holder and carrier was designed to locate the samples for irradiation in the desired location within the confines of the existing beamline infrastructure. The approach was validated by examining an irradiation time dependent increase in γH2AX foci formation or fluorescence intensity, with and without the presence of [^10^B]-BPA, by microscopy and flow cytometry in glioblastoma tissue cultures. These results show a statistically significant increase in DNA damage in cells treated with [^10^B]-BPA compared to neutron irradiation alone. This confirms that simple, low cost repurposing of existing beamlines is an effective method for establishing a basic neutron radiation biology capability and has resulted in the first thermal neutron source available for this kind of research in Australia.

## Materials and methods

This section describes the experimental procedures for neutron irradiation studies at the OPAL reactor. The methodology includes techniques for sample positioning using EBT3 radiochromic films with cadmium sheets to visualize neutron field distribution, and quantification of thermal neutron fluxes using gold neutron activation. The biological experiments utilized U87-MG and T98G glioma cell lines cultured in MEM media and treated with 500 μM [10B]-BPA prior to neutron irradiation at estimated fluences of 1.5 × 10^10^ n/cm2 and 1.5 × 10^11^ n/cm2. Post-irradiation analysis included immunofluorescence staining to quantify γH2AX foci formation, with visualization via fluorescence microscopy and quantitative assessment through both manual counting and flow cytometry.

### Sample positioning

The OPAL reactor supplies 15 instruments with neutron beams however, one of its key applications is manufacturing of radiopharmaceuticals and neutron transmutation doping (NTD) of silicon. This requires inserting assemblies into the reactor core, which change the neutron scattering properties and may alter the spatial distribution of the neutron beams entering the beam port. Thus, characterisation of the neutron field position is necessary before commencing any neutron capture experiment to assure consistent sample positioning. Halving the distance between the pinhole and the samples increases not only the neutron flux, but also the variability in neutron intensities across the field, which is particularly important when operating in the high-intensity mode. Therefore, it is necessary to develop a robust and quick method to evaluate the relative position and planar distribution of the beam at Dingo.

A simple solution for efficient estimation of the neutron field characteristics using EBT3 radiochromic films (Ashland) placed behind a 1.2 mm cadmium sheet was implemented. The cadmium sheet is wrapped in aluminium foil and the film affixed to it via aluminium adhesive tape. This assembly is attached to the sample holder, with the Cd facing the beam, placed into the relevant location in the beam path and irradiated for 10 min. Following irradiation, the cadmium sheet is removed leaving the film in place and left to self-develop for another 10 min to visualise the position of the beam relative to the sample holder. This approach enables the acquisition of an almost immediate relative image of the thermal neutron field through the low-energy γ-rays produced by neutron capture in cadmium which ensures samples are correctly positioned in the desired planar region of the field. Since the reaction rates in the polymer depend on the deposited energy, the initial optical signal drops with decreasing neutron flux incident on the cadmium sheet. Thus, to determine the semiquantitative profiles of the neutron beam intensity, the film can be removed and self-developed for ~ 12 h to account for the non-uniform response, as per manufacturer’s directions. Following this the film is scanned using a flatbed scanner to generate a 2D image. The optical density (OD) of the film is calculated from the pixel intensity which in turn is proportional to thermal neutron flux and can be plotted in ImageJ^16^. Percentage transmittance ($\%T$) is calculated as the fraction of the light that passes through the film, collected as grey values for each pixel. $\%T = (\frac{I}{Imax})x100$ where $I$ is the transmitted value through the film and $Imax$ is the maximum value of the incident light following transmission. OD was calculated by the relationship $OD = 2-log\%T$. Profiles of interest are extracted and the data normalised by expressing as the percentage of the mean OD.

### Thermal neutron flux

Thermal neutron fluxes were quantified by gold neutron activation technique using the methodology covered in Jakubowski et al*.*^10,17^. The gold wires were pre-weighed and positioned on 2 cm × 2 cm cassettes containing the biological samples that were arranged in a 3 × 3 grid. Samples were then aligned to the geometric centre of the beam using the aforementioned technique and irradiated for 8 h. The characteristic γ-ray emission of ^198^Au was measured with a high-purity Germanium detector (HPGe). In this work, we used bare gold wires adjacent to the samples and the average thermal to epithermal neutron ratios to determine the thermal neutron fluxes. These were previously measured with cadmium-paired gold foils distributed across the field in the same location at Dingo’s tertiary shutter^18^. These ratios were then used to estimate the epithermal neutron flux (above the cadmium cut-off), subtract it from the bare gold wire results, and determine the thermal neutron flux during the measurement. Further details can be found in Jakubowski et al.^18^.

### Cell culture and Irradiation

Two human glioblastoma cell lines, that are representative of many features typical to glioblastoma, were used for validation of this approach to in vitro neutron irradiation. U87-MG (CellBank Australia, 89081402) and T98G (CellBank Australia, 92090213) cells were propagated in Minimum Essential Media (MEM, Gibco 11095098), supplemented with 10%FBS, 100U/ml penicillin and 100 μg/ml streptomycin, at 37 °C in a 5% CO_2_ atmosphere. The day prior to irradiation 1 × 10^6^ cells were seeded into a 24 well, optical base, tissue culture treated, assay plate to achieve ~ 80% confluence. Cultures were treated with 500uM [^10^B]-BPA (> 98.4% ^10^B, Interpharma Praha, Prague, Czech Republic), which was dissolved into cell media over a 2 h time period at ~ 40 °C in an ultrasonic water bath, and left to incubate for 2 h. All the wells were filled with warm D-PBS to reduce headspace and then sealed with a sterile adhesive film. The sealed plates were placed in the vertical position on the sample holder mounted to a specially designed trolley (Fig. 2B). The sample was manoeuvred into the irradiation position (Fig. 2A) and irradiated for a period of 1 min or 10 min achieving an estimated fluence of 1.5 × 10^10^ n/cm^2^ and 1.5 × 10^11^ n/cm^2^ respectively.

### Immunofluorescence

Following irradiation, cells were incubated for 40 min at 37 °C, 5% CO_2_ after which they were fixed in 10% NBF and stored at 4 °C in PBS until processing. The U87-MG and T98G cells were washed with PBS, and permeabilized with 0.1% Triton-X for 10 min at room temperature on a rocker. Samples were blocked with 5% BSA for 1 h at room temperature on a rocker. Cells were then incubated with primary antibody (γH2AX anti-mouse (Merk Life Science, Australia) 1:1200 dilution in 1% BSA) overnight at 4 °C, washed with 0.1% PBST, and incubated with secondary antibody (Alexa Fluor 488 goat anti-mouse (AbCam, Australia) 1:800 dilution in 1% BSA) for 1 h at room temperature on a rocker. After a final PBST wash, cells were first counterstained with Phalloidin iFluor-594(1:1000, Abcam, Australia) for 1 h and DAPI (4′,6-diamidino-2-phenylindole) (1 µg/ml) for 20 min at room temperature, washed again, and mounted using FluoroShield mounting media (Sigma). Samples were left to cure overnight at room temperature (protected from light) and stored at 4 °C for up to two weeks prior to analysis.

The resulting γH2AX foci were quantified by counting the number of foci per nucleus in 55 randomly selected cells, by eye, using a 100 × objective. Images of foci were also taken using a 63 × objective. Focal stacks of representative regions were acquired using a Zeiss Imager Z2 microscope and deconvolved using the Zen deconvolution package. Maximum intensity projections were prepared from the deconvolved stacks to illustrate the distribution of foci within the nucleus.

### Flow cytometry

At 60 min post irradiation, cells were washed with PBS and incubated in non-enzymatic cell dissociation solution (Sigma #C5914) for 5 to 10 min and kept on ice.

Recovered cells were washed and stained with DAPI (0.1 μg/ml) followed by an additional 2 PBS washes. Cells were then permeabilized and stained with γH2AX (Phospho-Histone H2A.X (Ser139) (CR55T33), PE, eBioscience™) antibody using the BD (Becton–Dickinson) Pharmingen™ Transcription Factor Buffer Set (#562574) following manufacturers’ recommendations. Acquisition was performed on a 5 lasers BD FACSymphony™ A3 Cell Analyzer. A minimum of 10,000 DAPI^neg^ cells were acquired per sample. Analysis was carried out using FlowJo® software.

### Statistical analysis

The Mann–Whitney U test^19^ was employed to analyse differences between groups in visualised γH2AX foci data. Visual inspection of the data indicated that foci per nucleus exhibited a skewed distribution within these populations. This skewing likely results from the elevated background of spontaneous foci formation characteristic of cancer cells^20^. The Mann–Whitney U test was selected as it compares medians rather than means, is robust against non-normal distributions, and is sensitive to differences in distribution shape. The U statistic is computed as U = n₁n₂ + [n₁(n₁ + 1)/2] − R₁, where n₁ and n₂ are the sample sizes and R₁ is the sum of ranks for the first sample. For our sample sizes (n = 55 per group), the U statistic approximates a normal distribution, allowing for standard significance testing. This nonparametric approach provides appropriate insights into how these treatments differ in the creation of DNA double strand breaks, particularly in cases such as this where the assumptions of parametric tests cannot be satisfied.

## Results

### Beam targeting and quality

**Fig. 3:**
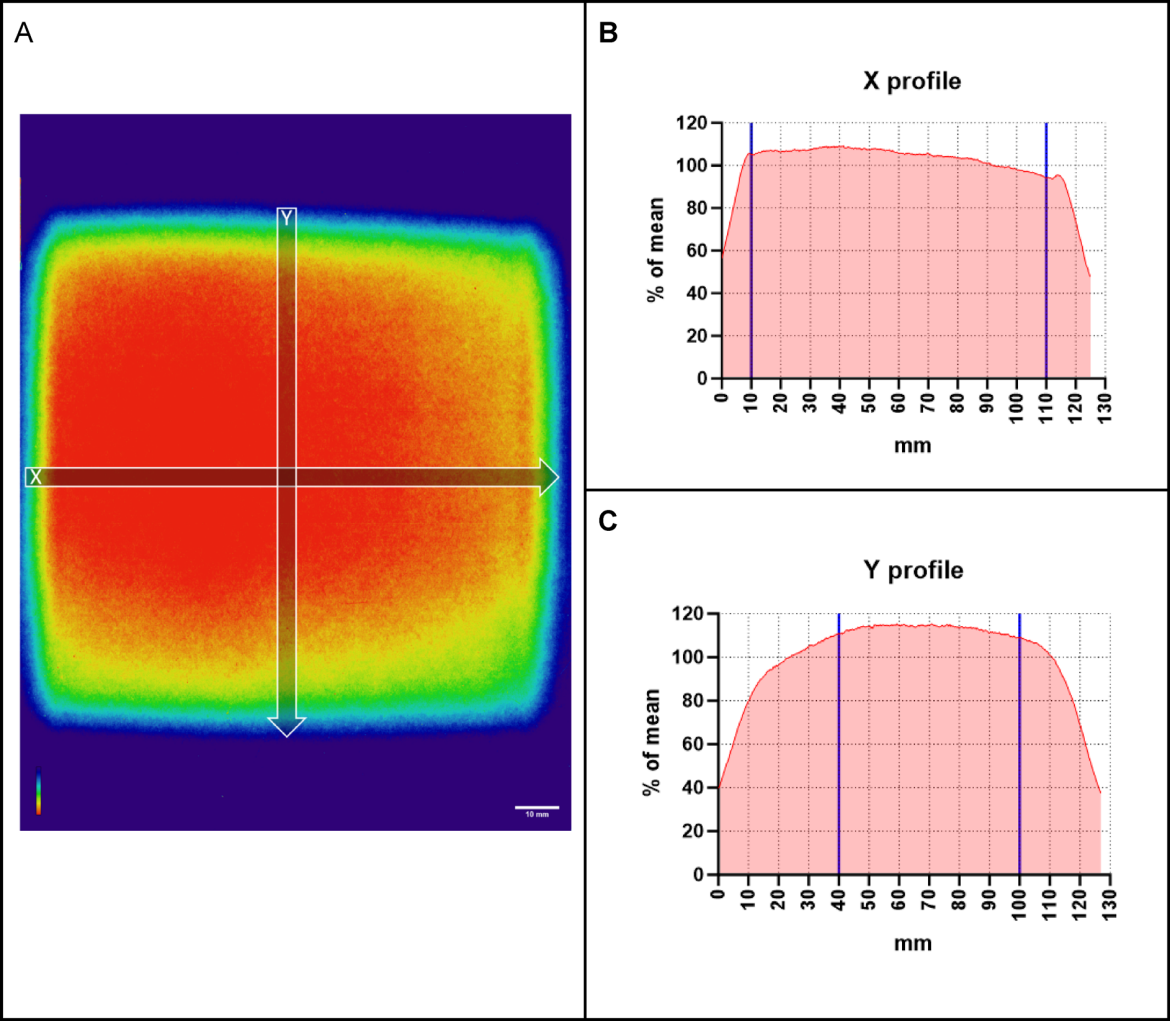
Thermal neutron field characteristics measured with the radiochromic film (**A**). The use of this method allows for a quick and accurate sample alignment with the beam. Line profiles, when taken through the centre, demonstrate the slightly skewed nature of thermal neutron flux within the beam. In the X profile (**B**) the beam intensity can be seen to drop off slightly from left to right but remains linear until it falls away sharply at the edge. The Y profile (**C**) shows a flatter profile but with much more pronounced shoulders. The proposed region for irradiation is delimited by the blue lines.

As previously demonstrated by Jakubowski et al., the neutron beam at Dingo constitutes approximately 59% thermal, 21% epithermal and 20% fast neutrons^17^. Imaging of the beam with radiochromic films shows an approximate square shape (Fig. 3). The beam profile data is presented as a percentage deviation from the mean. The x axis (left to right) shows a relatively flat profile with sharp shoulders. The coefficient of variation along the length of the profile is 12.15% with a skewness of − 2.4 indicating a left bias. The y axis (top to bottom) profile has rounded shoulders and a more pronounced drop off in thermal neutron flux towards the top of the beam. The coefficient of variation along its length is 19.6% with a skewness of − 1.6 indicating a slight bias to the top but overall more symmetrical than the x profile. This qualitative assessment indicates the need to consider a reduced area for the irradiation so as to improve the neutron flux homogeneity on the irradiated samples. By considering an area of 10 cm in the X axis and 6 cm in the Y axis, the % deviation from the mean is 3.7% and 1.5% respectively. Location in the tunnel is determined relative to the sample holder, allowing the samples to be reliably and reproducibly placed into this location.

**Fig. 4:**
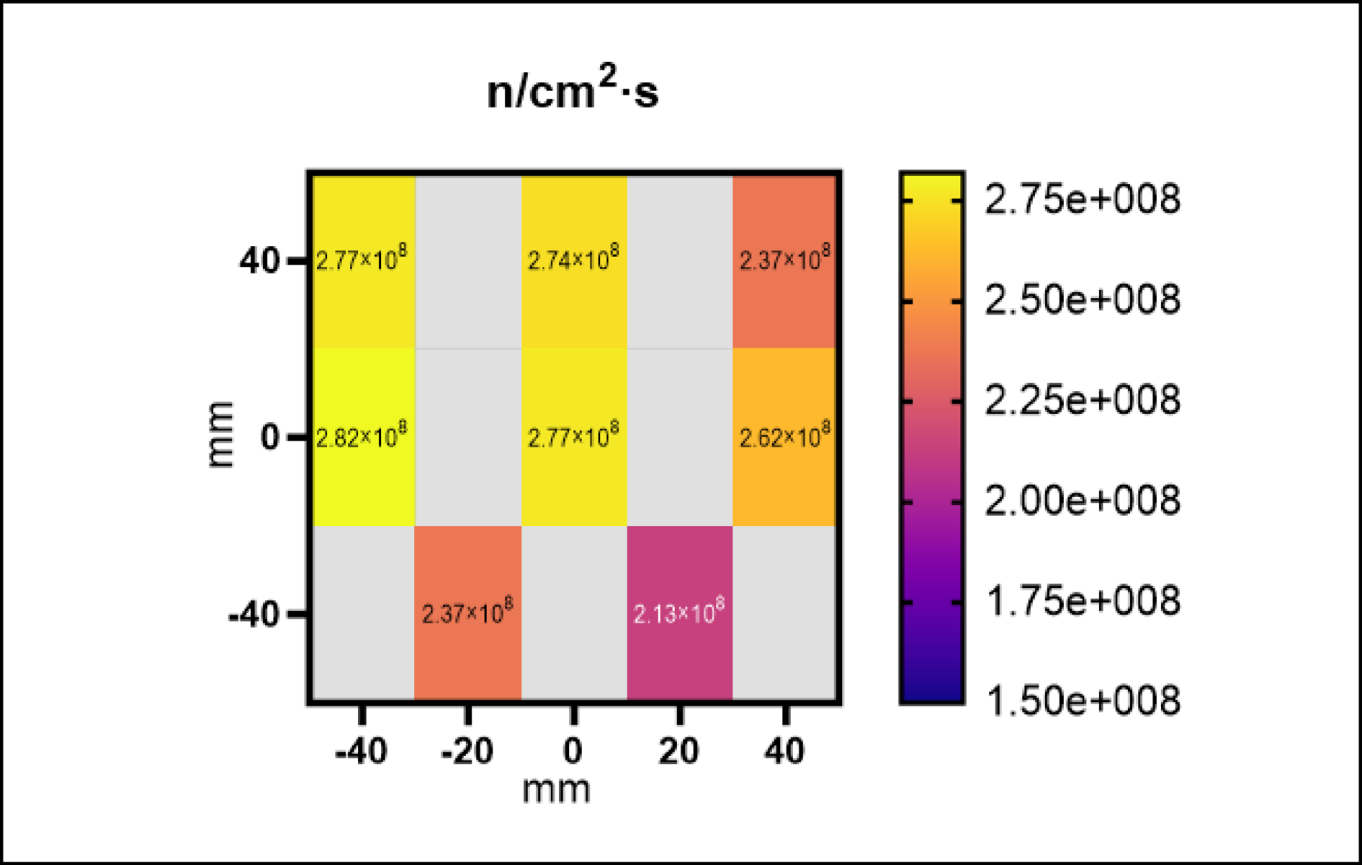
Spatial distribution of the thermal neutron field measured using gold wire activation technique. The variation in neutron flux across the field has been estimated to be approximately 2.73 × 10^7^ n/cm^2^ s.

The relative thermal neutron spatial distribution shows good agreement with the results of the absolute thermal neutron flux measurements using gold wires (Fig. 4). The 8 × 8 cm array, positioned in the centre of the beam, measured a neutron flux of 2.57 × 10^8^ n/cm^2^⋅s (± 2.73 × 10^7^).

### Radiobiological validation

**Fig. 5:**
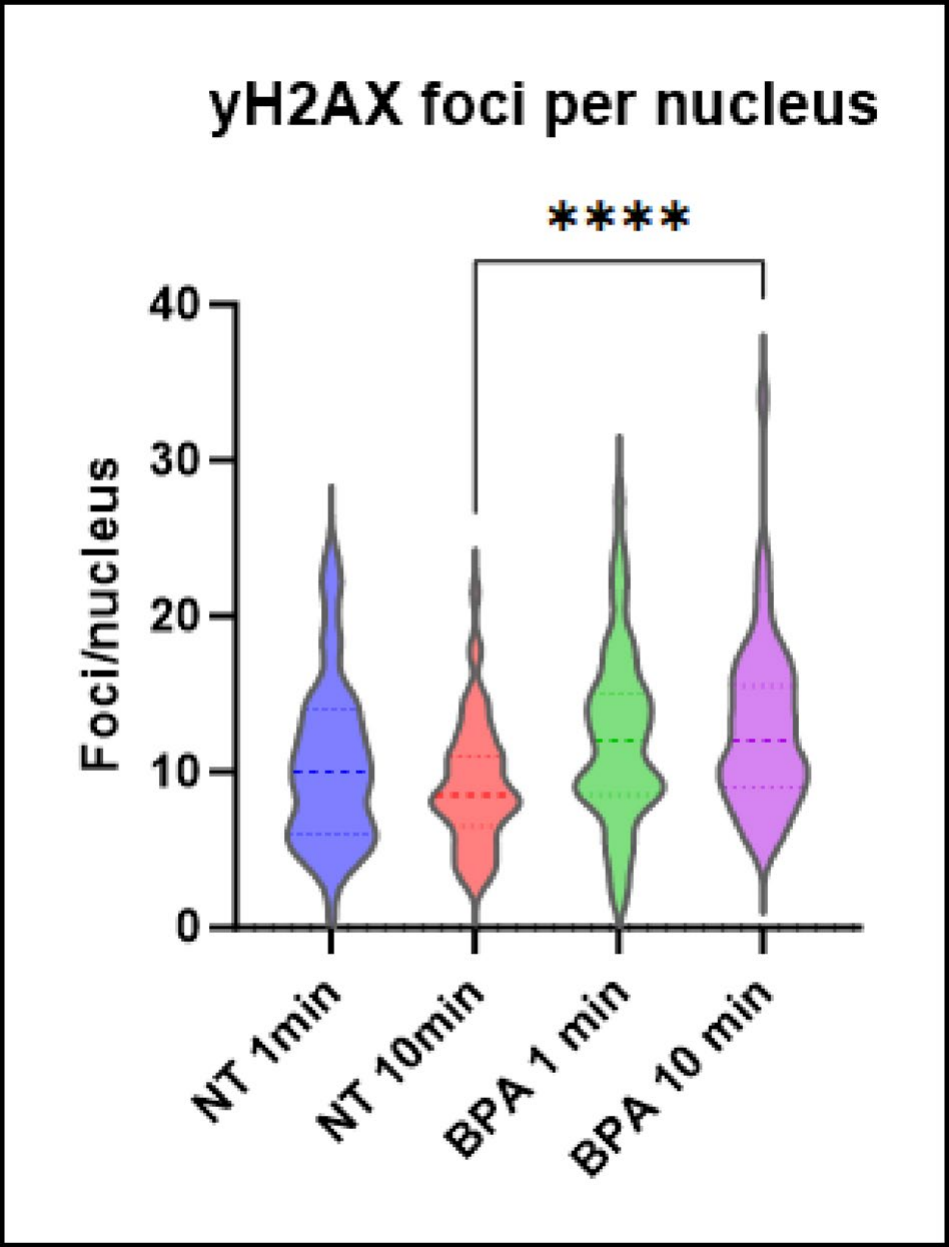
Number of γH2AX foci per nucleus in U87MG cells following 1 min and 10 min of neutron irradiation. Statistical analysis using the Mann–Whitney U test showed a significant difference between non-treated (NT) and 500 uM [^10^B]-BPA treated cells (1 min; *p* = 0.0010, 10 min; *p* < 0.0001). No significance is observed from the 1 min irradiation time in this cell line. This shows a measurable impact of neutron capture as a result of irradiation that is significantly increased from just neutron irradiation alone.

The number of γH2AX foci per nucleus increased significantly with the addition of 500 μM [10B]-BPA when compared to irradiation alone, demonstrating the presence of a neutron capture dose (Fig. 5). A Mann–Whitney U test was implemented to compare foci/nucleus between treatment groups. For the 10-min irradiation groups, there was a significant difference (U = 867.5, *p* < 0.0001) between cells without ^10^B-BPA (median = 8.5, 95% CI [8.11, 10.23], n = 55) and cells treated with ^10^B-BPA (median = 12.0, 95% CI [11.38, 14.26], n = 55). The 1 min irradiation groups did not show a statistically significant difference (U = 1226, *p* = 0.0868) between cells without ^10^B-BPA (median = 10.45, 95% CI [9.036, 11.87], n = 55) and cells treated with ^10^B-BPA (median = 12.18, 95% CI [10.68, 13.69], n = 55). The results obtained from this test appear to correlate visually with what is observed in the data distribution and indicate that the neutron capture dose incurred by the cells, with 500uM ^10^B-BPA and 10 min irradiation time (corresponding to a neutron fluence of 1.5 × 10^11^ n/cm^2^), has a significant impact on the number of double strand breaks to DNA.

Quantification of foci in this manner was complicated in these cell lines by the presence of a very high background of foci production, which can often be seen in cancer cell lines^20^. The background foci per nucleus of these cultures is around 10 and results in non-normally distributed data. This widely accepted technique of counting foci also has limitations at higher doses with the foci becoming too close together to reliably distinguish one from the other leading to inaccuracy in counting. While visual counting of foci was possible in U87MG cells the response generated in T98G cells requires a different approach.

**Fig. 6:**
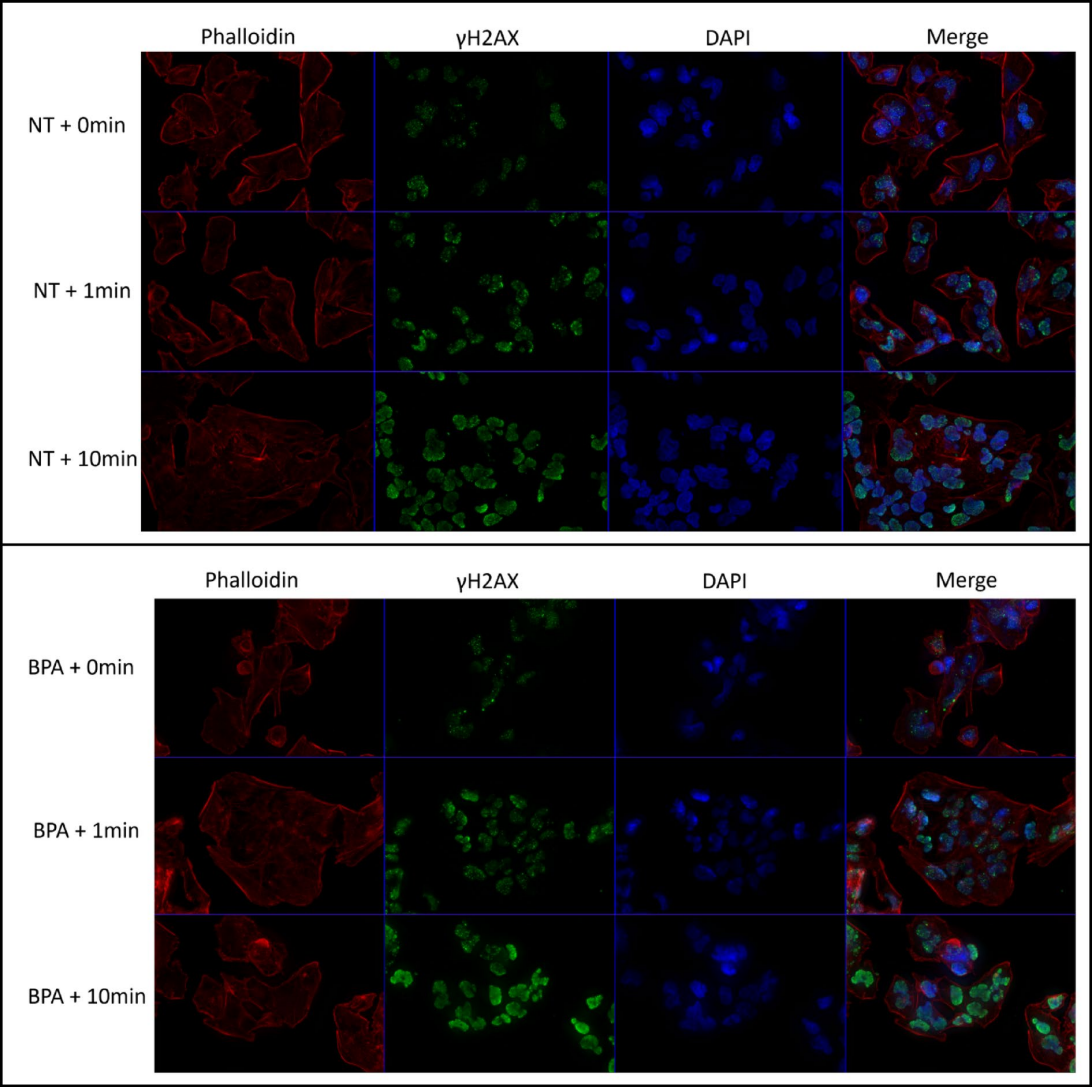
Immunocytochemistry analysis of γH2AX foci formation in T98G cells in response to neutron irradiation in the presence or absence of 500 μM [^10^B]-BPA. When [^10^B]-BPA was present, an increase in γH2AX foci formation was observed following 1 min of neutron irradiation. At 10 min, many cells began to exhibit a pan-nuclear response, which was not present in the cells that received no treatment (NT). This demonstrates that neutron capture by [^10^B]-BPA increases the formation of DNA double-strand breaks and is significantly more pronounced than neutron irradiation alone. The pan-nuclear response observed at 10 min post-irradiation indicates that the damage is extensive and will likely result in cell death.

Deconvolved z-stack images of these cultures demonstrate the nature of γH2AX staining observed during the counting procedure (Fig. 6). A visual increase in both foci number and fluorescence intensity of pan-nuclear staining (uniform staining of the nucleus) can be observed in T98G cells to correlate with the time of irradiation. Cells treated with 500 μM [^10^B]-BPA show increased levels of foci formation before tending to high levels of pan-nuclear staining following the 10 min irradiation.

**Fig. 7:**
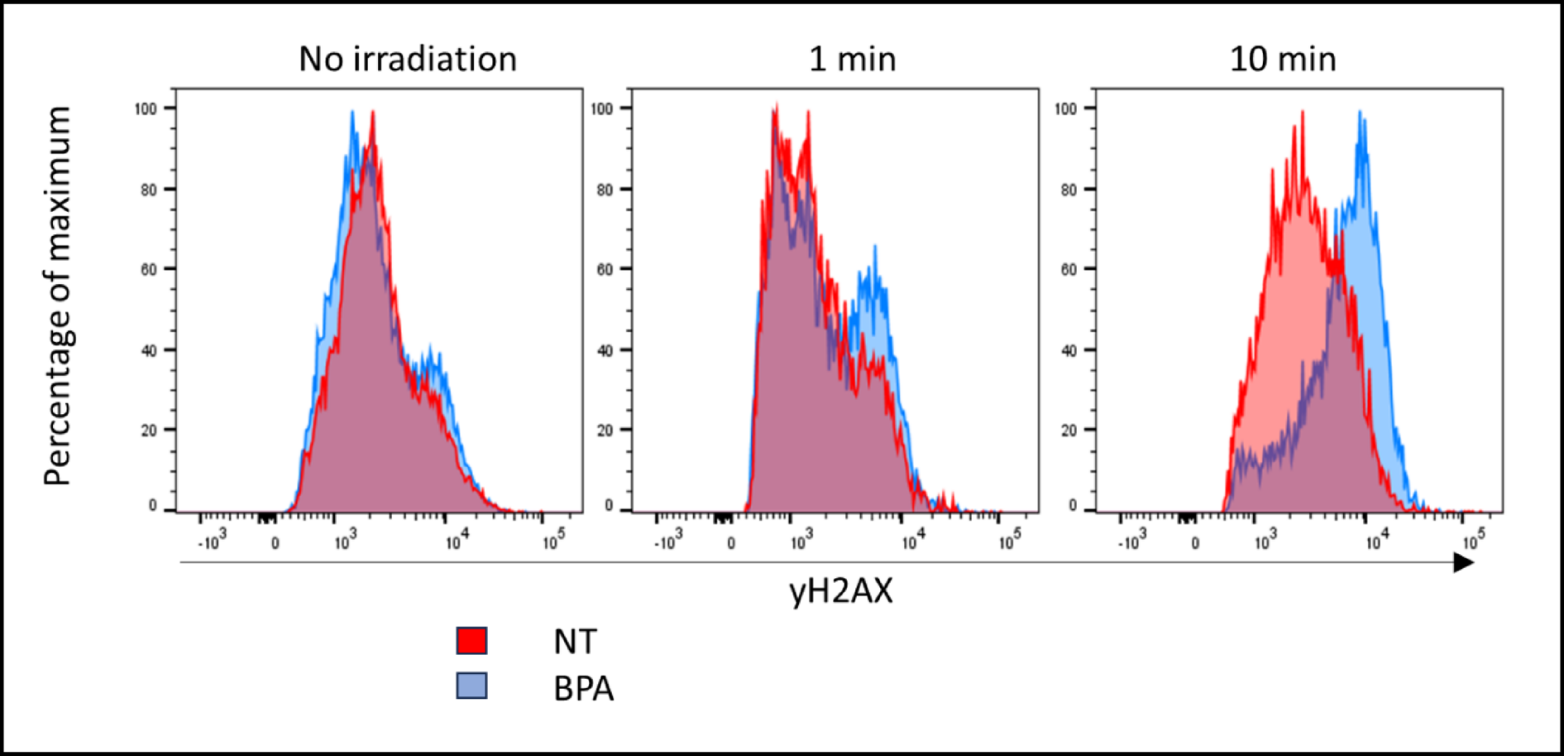
γH2AX staining flow cytometric analysis of T98G cells gated on live DAPI^−^ cells 60 minutespost neutron irradiation in the presence or absence of 500 μM [^10^B]-BPA. γH2AX staining intensity reveals a minor subset of cells with staining without neutron exposure with no detectable difference with or without BPA treatment. An increased exposure time to neutron irradiation correlates with an increased proportion of cells with increased γH2AXstaining intensity, with a majority of cells becoming highly positive for γH2AX following 10 minutesof neutron exposure when BPA is present.

For cells, such as T98G exhibiting pan-nuclear H2AX responses, counting foci is no longer a valid method of quantification. In this case this increase in fluorescence intensity along with foci formation can be investigated by flow cytometry (Fig. 7). It showed an irradiation-time-dependent increase in γH2AX staining intensity in response to neutrons following incubation with 500 μM [^10^B]-BPA with the majority of the cells exhibiting γH2AX staining in the cultures treated with [^10^B]-BPA following 10 min of neutrons. In contrast, only a minor increase in γH2AX staining intensity is detectable in NT controls following neutron irradiation.

## Discussion

To the best of our knowledge, this is the first, and only, neutron beam available for radiation biology research in Australia. The approach described above demonstrates that, within the restrictive environment of nuclear reactor infrastructure, repurposing of existing beamlines is a potentially viable approach for the establishment of a neutron radiation biology capability. While not comparable in capability, the cost of this approach is far less than building a dedicated facility. No permanent alterations to the existing infrastructure were required to achieve the successful irradiation of viable human tissue cultures at biologically significant flux and energy.

By utilising commercially available radiochromic films, we were able to efficiently evaluate potential changes to the relative position and spatial distribution characteristics of the thermal neutron component of the beam at Dingo, which can occur due to modifications of the reactor core configuration. This approach increases experimental reproducibility and improves the positional accuracy of the samples. The thermal neutron flux can be effectively measured using gold NAA technique.

Monte Carlo modelling of the neutron beam flux at the sample stage by Jakublowski et al. reported thermal neutron flux when running in high intensity mode at the sample stage of 4.7 × 10^7^ n/cm^2^⋅s^10^. This accounts for approximately 59% of the neutron beam while the epithermal and fast neutron components comprise 21% and 20%, respectively. By approximately halving the distance to the primary shutter, the thermal neutron flux increases by nearly the factor of 4. It can be further increased by placing the samples immediately behind the tertiary shutter entry reaching 2.52 × 10^8^ (± 2.73 × 10^7^) n/cm^2^ s. A previous experimental irradiation of tissue cultures by Kang et al.at the Dingo sample stage used the 4.7 × 10^7^ n/cm^2^⋅s flux to deliver 1.2 × 10^12^ n/cm^2^ over a 7 h time period^21^. Cells were kept in zero headspace at room temperature for the duration, which can introduce major confounding factors to the analysis of cell survival and proliferation, and precludes many measurements of interest such as the DNA damage measurement demonstrated here. Repeating this experiment with the methodology described above would result in a reduction of irradiation times to 79 min, with further reductions possible if the irradiation is restricted to the highest flux portion of the beam. This results in a much improved throughput and reduction of time spent at non-physiological conditions. Further opportunities for development to improve precision and accuracy, and therefore reduce irradiation time, have been identified. These include the integration of real-time quad-MOSFET beam-monitoring devices for instant feedback and automated instrument control based on the accumulated signal, rather than measurement time^14^.

Viable human tissue cultures were placed behind the tertiary shutter and irradiated for periods of up to 10 min; the cultures were successfully recovered and the resulting activation of HR and NHEJ DNA repair pathways measured by γH2AX immunocytochemistry and flow cytometry. Significant increases in DNA damage were observed from the inclusion of 500 μM ^10^B-BPA, demonstrating the successful application of thermal neutron radiation to these samples resulting in the delivery of an in vitro ^10^B neutron capture dose. While the maximum achievable flux of 2.52 × 10^8^ n/cm^2^ s is lower than minimum clinical BNCT recommendations of 5 × 10^8^ n/cm^2^ s, the significant increase in γH2AX response demonstrates that this flux is suitable for in vitro studies and drug development.

There are two major limitations that must be taken into consideration when irradiating tissue cultures as described above:

1. The horizontal geometry of the beam requires that tissue cultures be held in a vertical position and;
2. Irradiations are conducted under ambient conditions.

To address point 1 all irradiations occur within air tight sealed containers, such as a 24 well plate with an adhesive seal or a screw top flask. To prevent cultures dehydrating during setup and irradiation time all containers are prepared with as close to zero headspace as practically achievable. In order to reduce the impacts of both points it is important to minimise the time the cultures are exposed to these conditions. Efficient sample setup and retrieval is key to keeping the impacts of these factors consistent between irradiations. The significance of these factors needs to be taken into consideration on a per cell line basis for future work, as the impacts on downstream analysis will vary cell line to cell line, and with the measurement being taken.

Inclusion of temperature control to the irradiation setup would be a valuable addition to this technique as it would allow for maintaining conditions in the cell cultures that are closer to physiological. Compact heating devices, such as Peltier heat exchanges that can be thermostatically controlled are being investigated. The complexities of placing such devices into such a neutron rich environment need to be taken into consideration from a functional and longevity perspective as well as the potential activation of the components. The current irradiation setup is constructed entirely out of plastic which does not activate to any significant extent under these conditions.

The gold neutron activation analysis technique used in this study is the definitive method for calculating thermal neutron flux coming from nuclear reactors. As this technique directly measures the activation of ^198^Au via gamma ray spectrometry, the quantification of the thermal neutron flux is straightforward and robust. The technique itself is time-consuming compared to the short irradiation times of 1 min or less used for the irradiation of biological samples, with results being received a minimum of 24 h post experiment. It also requires sufficient gold activation for reliable quantification of the gamma ray emission and calculation of thermal neutron fluxes. The addition of a real-time neutron monitoring device that can distinguish between thermal and epithermal neutrons would be a very valuable addition. Simulation testing has shown promising results for a device using MOSFET detectors covered with various converter materials: boron carbide alone, a combination of cadmium and boron carbide, or polyethylene^14^. Since the neutron beam properties can vary between the measurements, real-time feedback will be extremely valuable to allow optimisation of experimental protocols to more efficiently utilise the available beamtime and improve both the precision, and accuracy of irradiations.

## Conclusion

The primary rate limiting factor for NCT research and development is the access to appropriate radiation sources. Typically, hours on such instruments are limited and the merit based access models extremely competitive. To accelerate development of novel NCAs and targets, more beamtime is required. We have implemented an approach to adapt Dingo, a thermal neutron imaging instrument on the OPAL reactor in Australia, to conduct biological irradiations for the purposes of radiobiological research and development. Sample positioning and techniques for beam targeting quality assurance and neutron flux measurements were implemented to provide a radiobiological irradiation capability with minimal modifications to existing infrastructure. Opportunities for further development to improve precision and accuracy have been identified. By promoting the exploration of existing nuclear reactor-based facilities for biological research, we aim to expedite NCA development and generation of radiobiological insights. This approach could enhance the application of more affordable accelerator-based neutron sources for cancer treatment by expanding the pharmacological options available for NCT and allow further optimisation of treatment plans. Additionally, particle beam therapy concepts like NCEPT—which utilise neutrons naturally generated during treatment—may further extend NCA applications to deep-seated and diffuse tumors, potentially introducing neutron capture therapy capabilities to established particle therapy clinics worldwide.

## Data Availability

All data generated and analysed in this publication is available from the corresponding author upon request.
